# Feasibility study on pre or postoperative accelerated radiotherapy (POP-ART) in breast cancer patients

**DOI:** 10.1186/s40814-020-00693-z

**Published:** 2020-10-10

**Authors:** Hans Van Hulle, Vincent Vakaet, Giselle Post, Annick Van Greveling, Chris Monten, An Hendrix, Koen Van de Vijver, Jo Van Dorpe, Pieter De Visschere, Geert Braems, Katrien Vandecasteele, Hannelore Denys, Wilfried De Neve, Liv Veldeman

**Affiliations:** 1 Department of Human Structure and Repair, Ghent University, Ghent, Belgium; 2 Department of Radiation Oncology, Ghent University Hospital, C. Heymanslaan 10, 9000 Ghent, Belgium; 3 Department of Pathology, Ghent University Hospital, C. Heymanslaan 10, 9000 Ghent, Belgium; 4 Department of Radiology and Nuclear Medicine, Ghent University Hospital, C. Heymanslaan 10, 9000 Ghent, Belgium; 5 Department of Gynaecology, Ghent University Hospital, C. Heymanslaan 10, 9000 Ghent, Belgium; 6 Department of Internal Medicine and Pediatrics, Ghent University, Ghent, Belgium; 7 Department of Medical Oncology, Ghent University Hospital, C. Heymanslaan 10, 9000 Ghent, Belgium

**Keywords:** Breast cancer, Neo-adjuvant radiotherapy, Neo-adjuvant chemotherapy

## Abstract

**Background:**

In early-stage breast cancer, the cornerstone of treatment is surgery. After breast-conserving surgery, adjuvant radiotherapy has shown to improve locoregional control and overall survival rates. The use of breast radiotherapy in the preoperative (preop) setting is far less common. Nevertheless, it might improve disease-free survival as compared to postoperative radiotherapy. There is also a possibility of downsizing the tumour which might lead to a lower need for mastectomy. There are some obstacles that complicate its introduction into daily practice. It may complicate surgery or lead to an increase in wound complications or delayed wound healing. Another fear of preop radiotherapy is delaying surgery for too long. At Ghent University Hospital, we have experience with a 5-fraction radiotherapy schedule allowing radiotherapy delivery in a very short time span.

**Methods:**

Twenty female breast cancer patients with non-metastatic disease receiving preop chemotherapy will be randomized between preop or postoperative radiotherapy. The feasibility of preop radiotherapy will be evaluated based on overall treatment time. All patients will be treated in 5 fractions of 5.7 Gy to the whole breast with a simultaneous integrated boost to the tumour/tumour bed of 5 × 6.2 Gy. In case of lymph node irradiation, the lymph node regions will receive a dose of 27 Gy in 5 fractions of 5.4 Gy. The total duration of therapy will be 10 to 12 days. In the preop group, overall treatment time is defined as the time between diagnosis and the day of last surgery, in the postop group between diagnosis and last irradiation fraction. Toxicity related to surgery, radio-, and chemotherapy will be evaluated on dedicated case-report forms at predefined time points. Tumour response will be evaluated on the pathology report and on MRI at baseline and in the interval between chemotherapy and surgery.

**Discussion:**

The primary objective of the trial is to investigate the feasibility of preop radiotherapy. Secondary objectives are to search for biomarkers of response and toxicity and identify the involved cell death mechanisms and the effect of preop breast radiotherapy on the in-situ immune micro-environment.

## Background

In early-stage breast cancer, the cornerstone of treatment is surgery: either mastectomy (ME) or breast-conserving surgery (BCS). After surgery, most patients receive some kind of adjuvant systemic therapy such as hormone therapy, chemotherapy (CT), targeted therapy, or a combination of these. After BCS, adjuvant radiotherapy (RT) has shown to improve locoregional control and overall survival rates. After ME, a benefit of adjuvant RT was observed only in node positive patients [1, 2]. In recent years, adjuvant or postoperative (postop) CT is increasingly replaced by preoperative (preop) or neo-adjuvant CT in patients with larger tumours to avoid mastectomy or tumours with a more aggressive phenotype (triple negative or HER2 amplified cancers) for early response assessment [3, 4]. Several randomized controlled trials demonstrated that there is no difference in overall survival whether CT is given pre- or postoperatively [5, 6].

The use of breast RT in the preop setting is far less common. It has been proposed for patients with inoperable or inflammatory breast cancer, and a recent retrospective study in breast cancer patients showed preop RT might improve disease-free survival as compared to postop RT [7]. In another retrospective study comparing preop versus postop radio- and chemotherapy, a possible benefit of preop treatment was suggested for tumours larger than 2 cm [8]. These benefits have also been observed in other cancer sites. There is evidence from randomized trials that preop RT is more effective than postop RT in patients with rectal carcinoma [9]. For soft tissue sarcoma, better local control rates have been described with preop than with postop RT [10]. From a radiobiological point of view, the benefits of giving RT preoperatively are obvious. In contrast to the postop setting, the vasculature is still intact and less radio-resistant tumour clones are present, both possibly increasing radiosensitivity. But there are other advantages of preop RT treatment such as improved delineation of the tumour and peritumoural bed for RT planning, which is evidently easier with the tumour still in place. In the postop setting, unnecessary larger volumes are delineated [11] and interobserver variability is larger [12] than in the preop setting. In preop RT, regions in need of higher doses can be better targeted. For the latter reason, less acute side effects and a better overall breast cosmesis is expected. There is also a possibility of downsizing the tumour which might lead to a lower need for mastectomy. While preop breast RT clearly has some advantages, there are some obstacles that complicate its introduction into daily practice. Preop RT therapy may complicate surgery or lead to an increase in postop wound complications or delayed wound healing. Whereas delayed wound healing compromises overall treatment time if adjuvant treatment is delivered postoperatively, this is not the case for preop chemo and radiotherapy.

Another fear of preop RT is delaying surgery and/or CT for too long, thus increasing the risk of distant metastases. However, this is not an issue if radiation courses are short. At Ghent University Hospital, we have experience with a 5-fraction RT schedule [13] allowing preop RT delivery in a very short time span. Large randomized trials confirm that moderate hypofractionation schemes in 15 or 16 fractions are at least equivalent in tumour control and toxicity although the biological equivalent total dose is lower than the traditional 50 Gy in 25 fractions [14–16]. Further acceleration to 5 fractions is expected to have an even greater radiobiological advantage concerning tumour control. In the UK FAST randomized trial, a schedule of 5 times 5.7 Gy, once a week, was compared to a normofractionation schedule of 25 times 2 Gy. Tumour control and toxicity were comparable after 3 and 8 years of follow-up [17]. In the UK Fast-Forward trial, a once-weekly 5-fraction schedule is studied. It may be considered for patients in whom a daily visit for 3 or 5 weeks is not acceptable however careful consideration of the dose per fraction is required [18]. At Ghent University Hospital (UZ Gent), a feasibility trial was started using the FAST scheme (5 × 5.7 Gy) over 12 days (instead of 5 weeks) in patients of 65 years or older. Additionally, patients requiring a boost received a simultaneously integrated boost to the tumour bed of 5 × 6.5 Gy. The final analysis on 95 patients shows < 10% grade 2–3 erythema, with only one case of moist desquamation, located at a skin fold [13]. With this RT schedule of 5 fractions in 12 days given preoperatively, we hypothesize that overall treatment time will not be increased.

Since the tumour is still present in preop RT, this presents a unique opportunity to identify the involved cell death mechanisms of breast RT. Classically, RT is considered to mediate its effects via the direct killing of cancer cells. It is now known that RT can induce systemic effects resulting in tumour responses outside the irradiated regions [19]. This phenomenon called the “abscopal”-effect has been reported in breast cancer [20] and several other kinds of malignancies [21] and is nowadays considered to be immune-mediated [22]. The hypothesis is that RT induces immunogenic cell death (ICD) through the release of tumour-associated antigens and damage associated molecular patterns (DAMPs), which leads to antigen uptake and dendritic cell maturation, resulting in the priming and clonal expansion of cytotoxic T-lymphocytes (CTLs) in the lymph nodes [23]. These CTLs then travel back to the tumour, becoming tumour-infiltrating lymphocytes (TILs). Radiation could increase these TILs in a clinical setting [24, 25] and, more importantly, a high level of (post-therapy) TILs is associated with a good prognosis [25–29]. Immunogenic cell death implicates the release of DAMPs and tumoural antigens through a disintegrated cell plasma membrane. The latter correlates with necrosis (regulated or secondary) instead of apoptosis, which was considered to be the principal mechanism of radiation induced cell death for years [30, 31]. Distinct cell death modalities may thus have a different (immunogenic) outcome.

Additional to the immunogenic cell death mechanism, this study will also investigate extracellular vesicles (EVs) as biomarkers for response and toxicity. EVs are nanometer-sized membrane vesicles that contain lipids, proteins, nucleic acids, and metabolites. Different cell types can release EV, including immune cells (monocytes, neutrophils, etc.), tumour cells, fibroblasts, and adipocytes (fat cells) [32, 33]. Extracellular vesicles are promising novel biomarkers because (1) their molecular content is a fingerprint of the releasing cells and their status and consists of proteins, lipids, and nucleic acids, (2) they are released in easily accessible body fluids such as blood, and (3) they are enriched for highly selected biomarkers which otherwise would constitute only a very small proportion (less than 0.01%) of the total molecular content of blood [34]. Analysis of Glypican-1 positive EV in circulation distinguishes with absolute specificity and sensitivity healthy subjects and patients with a benign pancreatic disease from patients with early- and late-stage pancreatic cancer [35, 36] and non-pancreatic cancer [37]. EV transfer from stromal to breast cancer cells regulates therapy resistance pathways [38]. miRNA levels in circulating EV identify remnant vital tumour tissue and are suitable to measure therapy response and relapse monitoring [39]. These pioneering studies suggest that quantification and characterization of EV can be implemented to predict therapy response.

## Methods

### Objectives

The primary objective of the trial is to investigate the feasibility of preop breast RT. Secondary objectives are to search for biomarkers of response and toxicity and identify the involved cell death mechanisms and the effect of preop breast RT on the in-situ immune micro-environment. The feasibility of preop RT will be assessed in terms of overall treatment time and toxicity related to surgery, CT, and RT. In the preoperative group, overall treatment time is defined as the time between diagnosis and the day of last surgery, in the postoperative group the time between diagnosis and last dose of RT. Secondary endpoints are the tumour response rate, the rate of mastectomy, identification of biomarkers of response and toxicity on EVs from plasma, immunohistochemistry of cell death markers and TILs on pre-treatment, post-RT as well as tumourectomy tissue samples, cardiac toxicity, lung function, and quality of life.

### Study population

**Fig. 1:**
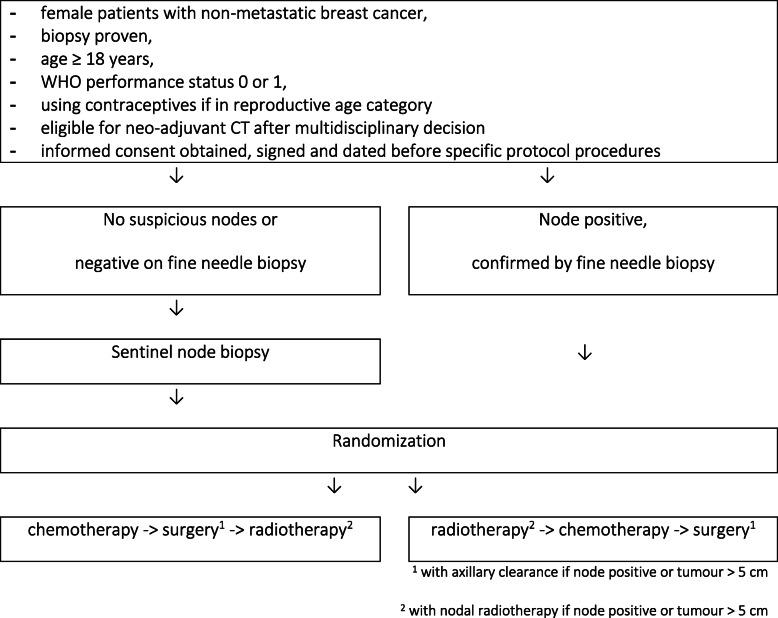
Flow chart

Twenty patients will be randomized between preop or postop RT as illustrated in Fig. 1. Inclusion criteria are female patients with non-metastasized breast cancer, for which a multidisciplinary decision must be made for preop CT, either this is for downsizing locally advanced breast cancer of because of type of tumour, such as triple-negative or HER2-positive early-stage breast cancer. Adjuvant hormone therapy will be administered to eligible women. For each patient, a biopsy with tumour histology, histological grade, ER/PR status, Her2/Neu status (amplification or not), and Ki67 status will be available. Exclusion criteria are distant metastases, inflammatory breast cancer (mastitis carcinomatosa), multifocal tumour, lobular carcinoma, bilateral breast cancer, history of cancer, with the exception of non-melanoma skin cancer, in situ cervix carcinoma, history of chemotherapy, history of radiation treatment, pregnant or breast feeding, or not using contraceptives if in reproductive age category, planned immediate reconstructive surgery, conditions making toxicity evaluation difficult (e.g. skin disorders), and amioderone treatment in the last 6 months. Exceptions to excluded carcinomas are made because these carcinomas occur frequently and result in a better life expectancy, so there is limited interference with the current study. There are also special exclusion criteria in function of chemotherapy, such as less than 2500 leukocytes or less than 1000/μL absolute neutrophil count.

In patients with clinically suspicious axillary lymph nodes a fine needle aspiration for cytology (FNAC) will be performed. If lymph node involvement is confirmed by FNAC, they will receive an axillary clearance after neoadjuvant treatment and axillary RT will be performed (either preop or postop). In clinically node-negative patients with a tumour of ≤ 5 cm, a sentinel node biopsy will be performed before the start of RT or CT. Patients with a tumour of > 5 cm, clinically node negative, will receive axillary clearance and axillary RT since a sentinel node biopsy is less reliable in these large tumours. Patients receiving postop RT will receive neoadjuvant CT followed by surgery (21–28 days after CT) and adjuvant RT starting 28–35 days after surgery. Patients receiving preop RT will receive RT first, followed by CT (5–8 days after the end of RT) and surgery (21–28 days after CT). In all patients, a marker clip will be placed in the tumour to determine its location before the start of any treatment. Ethics approval has received (EC2018/0599) and the study is registered at clinicaltrials.gov (NCT03783364).

### Treatment

Patients will be treated with 4 cycles of epirubicin and cyclophosphamide either in a dose dense scheme every 2 weeks or in a non-dose dense scheme every 3 weeks, followed by 12 weeks of paclitaxel. The 2 type of durations in chemotherapy makes the comparison of duration of treatment difficult, only the delay in treatment will be measured and not the normal duration of systemic therapy. For Her2 amplified tumours, trastuzumab will be added to the treatment concomitant with paclitaxel, every 3 weeks for a total of 18 cycles.

All patients will be treated according to routine practice at Ghent University Hospital. If lymph node irradiation is not indicated (i.e. patients with a negative sentinel node procedure), patients will be treated in the prone position if possible [40–42]. All other patients will receive treatment in the supine position. Left-sided breast cancer patients treated in the prone position will undergo two simulation CT’s: shallow breathing and deep inspirational breath hold (DIBH). This is a technique used to reduce heart dose by increasing the distance between the treated breast and the heart. Patients are asked to take a deep breath and block inspiration for a limited time span during which radiation is delivered. Only when the mean heart dose exceeds 0.73 Gy will the technique be used for radiation delivery. In all other cases, treatment will be delivered during shallow breathing [43]. All patients will be treated in 5 fractions of 5.7 Gy to the whole breast with a simultaneous integrated boost (SIB) to the tumour/tumour bed of 5 × 6.2 Gy. In case of lymph node irradiation, the lymph node regions will receive a dose of 27 Gy in 5 fractions of 5.4 Gy. Radiotherapy will be performed every other day, thus permitting cell repair in between fractions. The total duration of therapy will be 10–12 days. The whole breast and lymph node regions (in case of lymph node irradiation) are delineated based on the ESTRO/PROCAB guidelines [44]. The heart is delineated based on the guidelines provided by Feng et al. [45]. In the preop radiotherapy group, gross tumour volume (GTV) is delineated on the CT-simulation scan with guidance of MRI. The clinical target volume for boost irradiation (CTV_boost) includes the GTV with a margin of 5 mm around the GTV. Around the CTV, a planning target volume for the SIB (PTV_boost) is created by adding a margin of 5 mm. A median dose of 31 Gy (5 × 6.2 Gy) is prescribed to the PTV_boost with a dose fall off region of 1.5 cm around this PTV_boost, not extending outside the breast. The dose fall off region receives a minimum dose of 27.08 Gy with 95% receiving at least 27.9 Gy. In the postop radiotherapy group, CTV_boost will be delineated based on the surgical clips, the histology report, and all available pre-operative information (clinical investigation, imaging). Around the CTV_boost, a dose fall off region of 2 cm is defined. The dose fall off region receives a minimum dose of 27.08 Gy with 95% receiving at least 27.9 Gy.

### Evaluation of endpoints

The feasibility of preop RT will be evaluated based on overall treatment time. From a clinical point of view, it is not warranted that preop RT leads to an increase in the overall treatment time, since this may compromise locoregional control and survival. However, it is assumed that preop RT will shorten the overall treatment time by about 14 days (SD 9days) since the interval between RT and CT is shortened considerably. A difference of less than 14 days is not considered clinically relevant.

**Fig. 2:**
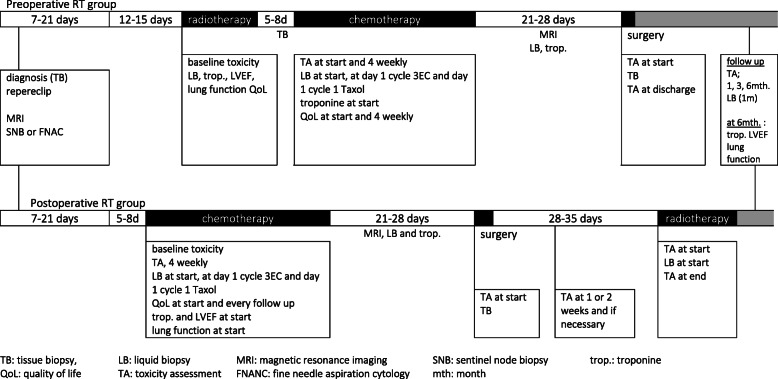
Study time table

As a start point for measuring the overall treatment time, diagnosis of breast cancer by biopsy is taken, while delays in treatment by decision-making can be taken in account. In the preop group, overall treatment time is defined as the time between diagnosis (biopsy) and the day of last surgery. In the postop group, overall treatment time is defined as the time between diagnosis (biopsy) and the last day of RT. Toxicity related to surgery, RT, and CT will be evaluated on dedicated case-report forms (in Appendix) at predefined time points as illustrated in Fig. 2. Tumour response will be evaluated on the pathology report (complete response rate and Pinder regression score) and on MRI at baseline and in the interval between CT and surgery.

To determine the mode of cell death evoked by pre-operative RT and its effect on the in situ immune micro-environment measurement (by immunohistochemistry (IHC)) of cell death markers and TILs will be performed on pre-treatment, post-RT (only in case of pre-operative RT), as well as tumourectomy tissue samples. The results of the IHC stainings for cell death markers will be correlated with the presence (or increase) of TILs in the same tissue samples and response to treatment.

All IHC stainings (cell death markers and TILs) will be performed on consecutive 3.5 μm slides of a representative formalin-fixed paraffin-embedded tissue block. A representative tissue block will be selected, taking into account the cellularity of the remaining tumour after RT (tissue biopsy after pre-operative RT), CT (tumourectomy post-op RT arm), or the combination of both (tumourectomy specimen pre-operative RT-arm). If residual tumour cells are absent, a tissue block with reactive changes (fibrosis, infiltration by foamy macrophages) will be selected. The following cell death markers will be examined: calreticulin (CRT), mobility group box 1 protein (HMGB-1), and Heat-Shock-Protein 70 (HSP70) for ICD; Cytokeratine 18 and caspase-3 for apoptosis; Senescense-associated β-galactosidase for senescence; phosphorylated mixed lineage kinase domain-like protein (pMLKL), and receptor-interacting protein kinase 3 (RIP3) for necroptosis and gluthatione peroxidase-4 (GPX-4) for ferroptosis.

HRQoL will be collected prospectively using different HRQoL instruments. For our analyses, only the items likely to be influenced by breast RT will be analysed. The European Organisation for Research and Treatment of Cancer (EORTC) 30-item Quality of Life Questionnaire (QLQ-C30) will be used, complemented by the breast cancer-specific module (QLQ-BR23). The EORTC QLC-C30 is a cancer-specific measuring instrument that describes five functional scales, three symptoms scales, six single-items scales, and a global health scale. Of these, we selected 2 functional scales (physical and social functioning), 2 symptoms scales (fatigue and pain) and the global health scale [46]. The EORTC QLQ-BR23, consists of 23 items, of which we included 2 symptom scales (i.e. arm symptoms and breast symptoms) and one functional scale (i.e. future perspective )[47]. The third questionnaire, the BREAST-Q questionnaire, was designed to evaluate outcome among women undergoing different types of breast surgery [48]. Breast satisfaction and physical well-being of the breast will be measured with respectively 6 and 7 questions. All questions of the BREAST-Q questionnaire will be used in this analysis. For all three questionnaires, a higher score indicates a better functioning for functional scales, while a higher score for symptom scales indicates a higher level of symptoms. The 3 questionnaires will be completed by the patient at 3 time points: before start of RT, 2 to 4 weeks after RT and 1 year after RT.

### Sample size and statistical analysis

While reduction of overall treatment time is the primary end point, sample size is made for this item. With 20 patients (10 patients in every treatment arm), a 14-day difference in overall treatment time can be detected with a power of > 90% (2-sided *t* test, α = 0.05).

The statistical package SPSS version 26 will be used to analyse the data. RT-related toxicity will be defined as any baseline toxicity that deteriorated during or after RT and any toxicity that arose during or after RT and was not present at baseline. A clinically relevant deterioration of HRQoL will be defined as a difference in score between baseline and 2–4 weeks after RT of 10 points or more. Differences in RT-related toxicity and clinically relevant deterioration of HRQoL between groups will be analysed by performing a chi-square test with a significance level of *p* < 0.05. For HRQoL, statistical differences between baseline scores and scores after 2 to 4 weeks will be evaluated with the Mann–Whitney *U* test. Due to the multiple tests for HRQoL, the Bonferroni correction will be used to avoid type I errors which leads to an adjusted *p* value of *p* < 0.005. 95% confidence intervals will be calculated. Ethics approval is received by the ethical board of University Hospital Ghent.

### Analysis of tissue and liquid biopsies

The results of the IHC stainings for these cell death markers will be correlated with profiles of CD8, CD4, CD3, CD68, and FOXP3 TILs (and the change in their presence after RT, CT or the combination of both). Patients will be divided into a high- and low-TIL group according to international guidelines.

Clinical (MRI) and pathological response assessed according to the Pinder regression grade (microscopic versus macroscopic disease)

Liquid biopsies (plasma samples) will be collected at consecutive time points (cfr. Fig. 2) to search for EV-associated biomarkers of response and toxicity. Blood will be collected in citrate tubes and platelet free plasma (PFP) will be prepared (2 × 2500×*g* centrifugation for 15 ) within 1 h after blood collection and stored at – 80 °C. EV will be isolated and characterized in compliance with MISEV2018, EV-TRACK, and Coumans et al. [49]. Chromatographic approaches will be combined with density gradient centrifugation to separate EV in two dimensions, size, and density, from contaminants such as lipoproteins, Argonaute-2 protein-miRNA complexes, and protein aggregates [50, 51]. Standard operating procedures have been optimized to enrich plasma EVs, to extract proteins/RNA, and to perform proteomics/small RNA sequencing. Currently, EVs from 6 ml of plasma allows to perform essential quality control experiments combined with proteomics and RNA sequencing [52]. Blood will be collected in citrate tubes and platelet free plasma (PFP) will be prepared (2 × 2500×*g* centrifugation for 15 min) within 1 h after blood collection and stored at – 80 °C. EVs will be isolated following SOPs, EV will be quantified by nanoparticle tracking analysis (NTA) and analysed by label-free mass spectrometry and RNA sequencing. We will implement receiver operating characteristics (ROC) to illustrate the performance of a biomarker, the sensitivity versus specificity, and will allow the selection of possible optimal EV biomarkers (number of EV and/or protein content and/or RNA content of EV). Advanced data analysis methods such as Perseus software [53] will further be used to enable comparison of expression levels within treatment groups and between treatment groups.

## Discussion

All types of treatment have an influence on general health-related quality of life [54]. A larger decrease in HRQoL due to RT is seen if patients started chemotherapy before or during RT [55–57]. As well as length of illness and treatment duration affect HRQoL negatively [58]. By integrating the boost and an accelerated RT in 5 fractions, overall treatment time can be reduced, with less acute [59] and 2 years toxicity [60] and better health-related quality of life [61]. Preop RT may reduce the overall treatment time with 2 weeks, which can lead to better quality of life for patients.

## Supplementary information

**Additional file 1.** Pre or postoperative accelerated radiotherapy (POP-ART)

## Data Availability

We do not wish to share our data. Our study protocol has undergone peer-review by the funding body.

## Abbreviations

BCS: Breast-conserving surgery
CRT: Calreticulin
CT: Chemotherapy
CTV: Clinical target volume
HMGB-1: Mobility group box 1 protein
HSP70: Heat-Shock-Protein 70
IHC: Immunohistochemistry
DIBH: Deep inspirational breath hold
EV: Extracellular vesicles
FNAC: Fine needle aspiration for cytology
GPX-4: Gluthatione peroxidase-4
GTV: Gross tumour volume
LB: Liquid biopsy
ME: Mastectomy
MRI: Magnetic resonance imaging
NTA: Nanoparticle tracking analysis
pMLKL: Phosphorylated mixed lineage kinase domain-like protein
preop: Preoperative
PTV: Planning target volume
QoL: Quality of life
RIP3: Receptor-interacting protein kinase 3
ROC: Receiver operating characteristics
RT: Radiotherapy
SIB: Simultaneous integrated boost
SNB: Sentinel node biopsy
TA: Toxicity assessment
TB: Tissue biopsy
TIL: Tumour-infiltrating lymphocytes
trop.: Troponine
