# Supplementary material for: Feasibility study on pre or postoperative accelerated radiotherapy (POP-ART) in breast cancer patients
Source: Pilot Feasibility Stud. 2020 Oct 10;6:154. doi: 10.1186/s40814-020-00693-z (PMC7547514; doi:10.1186/s40814-020-00693-z)
Supplement: Supplementary file 1 — Additional file 1. Pre or postoperative accelerated radiotherapy (POP-ART) [file 40814_2020_693_MOESM1_ESM.zip › Additional file 1/POP-ART baseline EN.docx]

Pre or postoperative accelerated radiotherapy (POP-ART)

Baseline evaluation form

Patient Initials ⬜ ⬜ ⬜ ⬜.

Date of Birth (dd/mm/yyyy) ⬜ ⬜ / ⬜ ⬜ / ⬜ ⬜ ⬜ ⬜

Date Completed (dd/mm/jjjj) ⬜ ⬜ / ⬜ ⬜ / ⬜ ⬜ ⬜ ⬜

Name + Signature of Person completing the CRF __________________________________

**Patient characteristics**

| length ⬜,⬜ ⬜ m  weight ⬜ ⬜ ⬜,⬜ kg  bra size (eg. 75 B) ⬜ ⬜ ⬜ . ⬜ | | menopause | ⬜ premenopausal  (regular menses)  ⬜ postmenopausal (>50y,  ≥ 1y of amenorrhea)  ⬜ none of the above or  hysterectomy or intra-  uterine device^[[1]](#footnote-1)^ |
| --- | --- | --- | --- |
| use of hormonal substitution therapy | ⬜ yes  ⬜ no | how many years ⬜ ⬜. | |

**Habits**

| Smoking | ⬜ never  ⬜ current  ⬜ former | | If applicable, years since stopped smoking | ⬜.⬜ | | Cigarettes per day  years smoking | | ⬜ ⬜ ⬜.  ⬜ ⬜. | | |
| --- | --- | --- | --- | --- | --- | --- | --- | --- | --- | --- |
| Alcohol | ⬜ never  ⬜ current  ⬜ former | Alcoholic consumptions/ week | | | before  diagnosis | | ⬜. | | after  diagnosis | ⬜. |

Habit comment: _____________________________________________________________________

**History Co-morbidity**

| **Genetics**  **Family history** | ⬜ no mutations found  ⬜ BRCA1+  ⬜ BRCA2+  ⬜ other: ____________________  ⬜ unkown (not tested)  ⬜ none  ⬜ breast cancer  ⬜ overian cancer  ⬜ other: ____________________ | Cardiovascular  Thyroid dysfunction  Diabetes  Rheumatological  Other | ⬜ no  ⬜ yes, _______________  ⬜ no  ⬜ yes, _______________  ⬜ no  ⬜ yes, _______________  ⬜ no  ⬜ yes, _______________  ⬜ no  ⬜ yes, _______________ |
| --- | --- | --- | --- |

**Medications**

____________________________________________________________________________________________________________________________________________________________________

**Tumour characteristics**

| size on MRI | | ⬜ ⬜ ⬜.mm | | | grade | | ⬜ I  ⬜ II  ⬜ III | | | |
| --- | --- | --- | --- | --- | --- | --- | --- | --- | --- | --- |
| cTNM stage | | | ⬜. | T (Tis, 1a-c, 2, 3, 4a-d, X) | | ⬜. | | N (0-3, X) | ⬜. | M (0,1-X) |
| Ki-67 status (%) | | | ⬜.⬜ ⬜ | | | ER status | | ⬜ positive  allred score ⬜. /8  percentage ⬜ ⬜ ⬜.  ⬜ negative  ⬜ unkown | | |
| HER-2 status | Immuno-histochemistry | | | ⬜ 1+  ⬜ 2+  ⬜ 3+  ⬜ 0  ⬜ unkown | |  |  |  |  |  |
|  |  |  |  |  |  | PR status | | ⬜ positive  all red score ⬜. /8  percentage ⬜ ⬜ ⬜.  ⬜ negative  ⬜ unkown | | |
|  | FISH/SISH/CISH | | | ⬜ amplification  ⬜ no amplification  ⬜ unkown | |  |  |  |  |  |

**Baseline evaluation for toxicities**

**Breast symptoms (side to be irradiated)**

**Pain** ⬜ none

⬜ only on contact

⬜ not only on contact, but occasionally

⬜ not only on contact and regularly

⬜ need for pain medication: __________________________

**Sense of heaviness** ⬜ yes ⬜ no

**Itching** ⬜ none ⬜ occasionally ⬜ regularly

**Arm symptoms (side to be irradiated)**

**Pain** ⬜ none ⬜ occasionally ⬜ regularly

**Sense of heaviness** ⬜ yes ⬜ no

**Shoulder pain (side to be irradiated)**

**Pain** ⬜ none ⬜ occasionally ⬜ regularly

**Impaired mobility**  ⬜ yes ⬜ no

**Pain other than breast, shoulder or arm pain**

⬜ none ⬜ occasionally ⬜ regularly

**Please indicate all painful localizations on the figure**


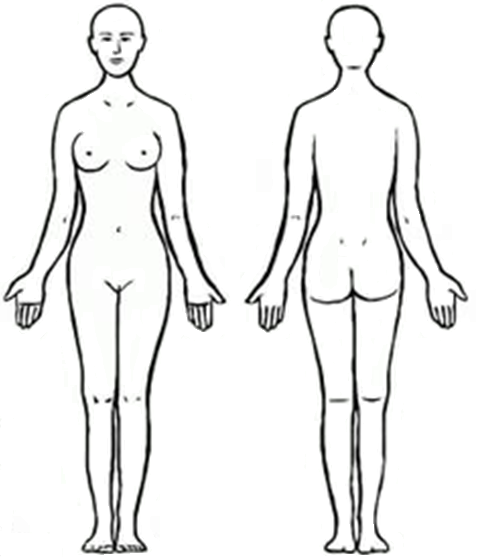


**Dysphagia according to the CTCAEE v. 4.03**

⬜ None

⬜ Symptomatic, able to eat regular diet

⬜ Symptomatic and altered eating/swallowing

⬜ Severely altered eating/swallowing; tube feeding or TPN or hospitalization indicated

⬜ Life-threatening consequences, urgent intervention indicated

**Dyspnea according to the CTCAE v. 4.03**

⬜ None

⬜ Shortness of breath with moderate exertion

⬜ Shortness of breath with minimal exertion; limiting instrumental ADL

⬜ Shortness of breath at rest; limiting self-care ADL

⬜ Life-threatening consequences, urgent intervention indicated

**Cough according to the CTCAE v. 4.03**

⬜ None

⬜ Mild symptoms, nonprescription intervention indicated

⬜ Moderate symptoms, medical intervention indicated; limiting instrumental ADL

⬜ Severe symptoms; limiting self-care ADL

**Fatigue according to the CTCAE v. 4.03**

⬜ None

⬜ Fatigue relieved by rest

⬜ Fatigue not relieved by rest, limiting instrumental ADL

⬜ Fatigue not relieved by rest, limiting self-care ADL

**Brachial plexopathy^[[2]](#footnote-2)^**

| Some change in the sensitivity of the arm or hand (on the side to be irradiated) | ⬜ yes  ⬜ no |
| --- | --- |
| Deaf feeling or tingling in the hand or fingers (on the side to be irradiated) | ⬜ yes  ⬜ no |
| Problems with carrying or lifting objects with the arm (on the side to be irradiated) | ⬜ yes  ⬜ no |
| Problems with fingers such as writing or loosening a bottle cap (on the side to be irradiated) | ⬜ yes  ⬜ no |

**Breast edema**

⬜ none

⬜ swelling or obscuration of anatomic architecture on close inspection

⬜ Readily apparent obscuration of anatomic architecture, obliteration of skin folds; readily apparent

deviation from normal anatomic contour, limiting instrumental ADL

⬜ Gross deviation from normal anatomic contour, limiting self-care ADL

**Color change breast**

⬜ None ⬜ Hyperpymentation ⬜ Hypopygmentation

**Color change areola**

⬜ None ⬜ Hyperpygmentation ⬜ Hypopygmentation

**Color change in de region around collarbone / armpits**

⬜ None ⬜ Hyperpygmentation ⬜ Hypopygmentation

**Fibrosis tumorbed (LENT-SOMA)**

⬜ none ⬜ Definite increased density and firmness

⬜ Barely palpable increased density ⬜ Very marked density, retraction and fixation

**Fibrosis outside tumorbed (LENT-SOMA)**

⬜ none ⬜ Definite increased density and firmness

⬜ Barely palpable increased density ⬜ Very marked density, retraction and fixation

**Telangiectasia tumorbed (LENT-SOMA)**

⬜ none ⬜ < 1 per cm^2^ ⬜ 1-4 per cm^2^ ⬜ > 4 per cm^2^

**Telangiectasia outside tumorbed (LENT-SOMA)**

⬜ none ⬜ < 1 per cm^2^ ⬜ 1-4 per cm^2^ ⬜ > 4 per cm^2^

**Dermatitis/desquamation according to the CTCAE v. 4.03**

0 = none

1 = Faint erythema or dry desquamation

2 = Moderate to brisk erythema; moderate edema; patchy moist desquamation, mostly confined to skin folds and creases^[[3]](#footnote-3)^

3 = Moist desquamation in areas other than skin folds and creases; bleeding induced by minor trauma or abrasion^2^

4 = Life-threatening consequences; skin necrosis or ulceration of full thickness dermis; spontaneous bleeding from involved site; skin graft indicated^2^


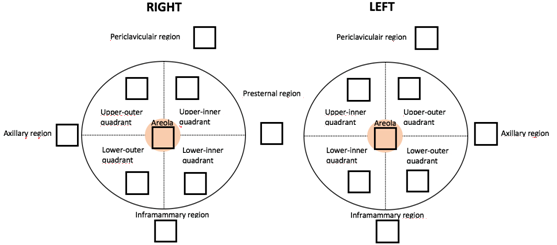


**Desquamation**

0 = None 1 = Dry desquamation 2 = Moist desquamation^2^

**
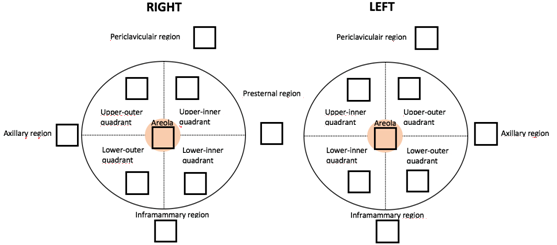
**

| **Arm circumference** | LEFT arm | RIGHT arm |
| --- | --- | --- |
| 15 cm above medial epicondyle | ⬜ ⬜, ⬜ cm | ⬜ ⬜, ⬜ cm |
| 15cm below medial epidcondyle | ⬜ ⬜, ⬜ cm | ⬜ ⬜, ⬜ cm |

**Brachial plexopathy, clinical examination:^[[4]](#footnote-4)^**

Flexion elbow

⬜ muscle can move joint against full resistance applied by examiner

⬜ muscle can move joint against mild/moderate resistance applied by examiner

⬜ muscle can move joint against gravity but without any resistance

⬜ muscle can move joint only if the force of gravity is eliminated

⬜ muscle contraction is seen or palpated but is insufficient to produce joint motion

⬜ no muscle contraction is seen or identified with palpation

Extension elbow

⬜ muscle can move joint against full resistance applied by examiner

⬜ muscle can move joint against mild/moderate resistance applied by examiner

⬜ muscle can move joint against gravity but without any resistance

⬜ muscle can move joint only if the force of gravity is eliminated

⬜ muscle contraction is seen or palpated but is insufficient to produce joint motion

⬜ no muscle contraction is seen or identified with palpation

Dorsiflexion wrist

⬜ muscle can move joint against full resistance applied by examiner

⬜ muscle can move joint against mild/moderate resistance applied by examiner

⬜ muscle can move joint against gravity but without any resistance

⬜ muscle can move joint only if the force of gravity is eliminated

⬜ muscle contraction is seen or palpated but is insufficient to produce joint motion

⬜ no muscle contraction is seen or identified with palpation

Plantar flexion wrist

⬜ muscle can move joint against full resistance applied by examiner

⬜ muscle can move joint against mild/moderate resistance applied by examiner

⬜ muscle can move joint against gravity but without any resistance

⬜ muscle can move joint only if the force of gravity is eliminated

⬜ muscle contraction is seen or palpated but is insufficient to produce joint motion

⬜ no muscle contraction is seen or identified with palpation

Shoulder abduction

⬜ muscle can move joint against full resistance applied by examiner

⬜ muscle can move joint against mild/moderate resistance applied by examiner

⬜ muscle can move joint against gravity but without any resistance

⬜ muscle can move joint only if the force of gravity is eliminated

⬜ muscle contraction is seen or palpated but is insufficient to produce joint motion

⬜ no muscle contraction is seen or identified with palpation

**Change in sensation (tested by gently rubbing)**

| 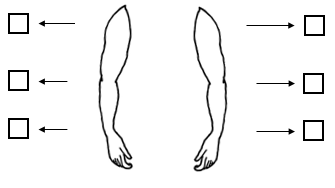 | 0 = no  1 = sensation different than the contralateral side  2 = no sensation at all |
| --- | --- |

**Myelosuppression**

leukocytes ⬜ ⬜ ⬜ ⬜ ⬜ ⬜./mm³

erythrocytes ⬜ ⬜ ⬜ ⬜./mm³

platelets ⬜ ⬜ ⬜ ⬜ ⬜ ⬜./mm³

**Gastro-intestinal complaints**

Constipation

⬜ None

⬜ Occasional or intermittent symptoms; occasional use of stool softeners, laxatives, dietary modification

⬜ Persistent symptoms with regular use of laxatives or enemas; limiting instrumental ADL

⬜ Obstipation with manual evacuation indicated; limiting self care ADL

⬜ Life-threatening consequences; urgent intervention indicated

Diarrhea

⬜ None

⬜ Increase of <4 stools per day over baseline; mild increase in ostomy output compared to baseline

⬜ Increase of 4 - 6 stools per day over baseline; moderate increase in ostomy output compared to baseline

⬜ Increase of >=7 stools per day over baseline; incontinence; hospitalization indicated; severe increase in

ostomy output compared to baseline; limiting self care ADL

⬜ Life-threatening consequences; urgent intervention indicated

Nausea

⬜ None

⬜ Loss of appetite without alteration in eating habits

⬜ Oral intake decreased without significant weight loss, dehydration or malnutrition

⬜ Inadequate oral caloric or fluid intake; tube feeding, TPN, or hospitalization indicated

Vomiting

⬜ None

⬜ 1 - 2 episodes (separated by 5 minutes) in 24 hrs

⬜ 3 - 5 episodes (separated by 5 minutes) in 24 hrs

⬜ >=6 episodes (separated by 5 minutes) in 24 hrs; tube feeding, TPN or hospitalization indicated

⬜ Life-threatening consequences; urgent intervention indicated

**Antibiotics intake in the last 2 weeks?**

| ⬜ yes  ⬜ no | If yes, reason: | _____________________________________________________________  _____________________________________________________________ |
| --- | --- | --- |

**Mucositis**

⬜ None

⬜ Asymptomatic or mild symptoms; intervention not indicated

⬜ Moderate pain; not interfering with oral intake; modified diet indicated

⬜ Severe pain; interfering with oral intake

⬜ Life-threatening consequences; urgent intervention indicated

**Hematoma/seroma in the treated breast**

⬜ None

⬜ Mild symptoms; intervention not indicated

⬜ Minimally invasive evacuation or aspiration indicated

⬜ Transfusion, radiologic, endoscopic, or elective operative intervention indicated

⬜ Life-threatening consequences; urgent intervention indicated

1. Need of plasma value: LH, FSH, estradiol [↑](#footnote-ref-1)
2. If brachial plexopathy is suspected, an electromyography should be performed [↑](#footnote-ref-2)
3. Document with photographs: an overview of the upper body (without the head) with the hands resting on the hips and one with the arms above the head and detailed snapshots of the areas of greatest toxicity. [↑](#footnote-ref-3)
4. If brachial plexopathy is suspected, an electromyography should be performed [↑](#footnote-ref-4)
