# Supplementary material for: Feasibility study on pre or postoperative accelerated radiotherapy (POP-ART) in breast cancer patients
Source: Pilot Feasibility Stud. 2020 Oct 10;6:154. doi: 10.1186/s40814-020-00693-z (PMC7547514; doi:10.1186/s40814-020-00693-z)
Supplement: Supplementary file 1 — Additional file 1. Pre or postoperative accelerated radiotherapy (POP-ART) [file 40814_2020_693_MOESM1_ESM.zip › Additional file 1/POP-ART chemotherapy - EN.docx]

Pre or postoperative accelerated radiotherapy (POP-ART)

CRF: Chemotherapy

Patient initials ⬜ ⬜ ⬜ ⬜.

Birth date (dd/mm/yyyy) ⬜ ⬜ / ⬜ ⬜ / ⬜ ⬜ ⬜ ⬜

Date Completed (dd/mm/yyyy) ⬜ ⬜ / ⬜ ⬜ / ⬜ ⬜ ⬜ ⬜

Name + Signature of Person completing the CRF __________________________________

| time of evaluation | 🞏 at EC1 | 🞏 at EC3 | 🞏 at Tax1 | 🞏 at Tax5 | 🞏 at Tax9 | 🞏 other |
| --- | --- | --- | --- | --- | --- | --- |

**Myelosuppression**

leukocytes ⬜ ⬜ ⬜ ⬜ ⬜ ⬜./mm³

erythrocytes ⬜ ⬜ ⬜ ⬜./mm³

platelets ⬜ ⬜ ⬜ ⬜ ⬜ ⬜./mm³

**Gastro-intestinal complaints**

Constipation

⬜ none

⬜ Occasional or intermittent symptoms; occasional use of stool softeners, laxatives, dietary modification

⬜ Persistent symptoms with regular use of laxatives or enemas; limiting instrumental ADL

⬜ Obstipation with manual evacuation indicated; limiting self care ADL

⬜ Life-threatening consequences; urgent intervention indicated

Diarrhea

⬜ none

⬜ Increase of <4 stools per day over baseline; mild increase in ostomy output compared to baseline

⬜ Increase of 4 - 6 stools per day over baseline; moderate increase in ostomy output compared to baseline

⬜ Increase of >=7 stools per day over baseline; incontinence; hospitalization indicated; severe increase in

ostomy output compared to baseline; limiting self care ADL

⬜ Life-threatening consequences; urgent intervention indicated

Nausea

⬜ none

⬜ Loss of appetite without alteration in eating habits

⬜ Oral intake decreased without significant weight loss, dehydration or malnutrition

⬜ Inadequate oral caloric or fluid intake; tube feeding, TPN, or hospitalization indicated

Vomiting

⬜ none

⬜ 1 - 2 episodes (separated by 5 minutes) in 24 hrs

⬜ 3 - 5 episodes (separated by 5 minutes) in 24 hrs

⬜ >=6 episodes (separated by 5 minutes) in 24 hrs; tube feeding, TPN or hospitalization indicated

⬜ Life-threatening consequences; urgent intervention indicated

**Allergic reactions since previous evaluation**

⬜ None

⬜ Transient flushing or rash, drug fever <38 degrees C (<100.4 degrees F); intervention not indicated

⬜ Intervention or infusion interruption indicated; responds promptly to symptomatic treatment (e.g.,

antihistamines, NSAIDS, narcotics); prophylactic medications indicated for <=24 hrs

⬜ Prolonged (e.g., not rapidly responsive to symptomatic medication and/or brief interruption of infusion);

recurrence of symptoms following initial improvement; hospitalization indicated for clinical sequelae (e.g.,

renal impairment, pulmonary infiltrates)

⬜ Life-threatening consequences; urgent intervention indicated

**Fatigue**

⬜ None

⬜ Fatigue relieved by rest

⬜ Fatigue not relieved by rest; limiting instrumental ADL

⬜ Fatigue not relieved by rest, limiting self care ADL

**Hospital admission since previous evaluation**

| ⬜ yes  ⬜ no | If yes, reason: | _______________________________  _______________________________ | number of days in hospital | □ |
| --- | --- | --- | --- | --- |

**Antibiotics required since previous evaluation?**

| ⬜ yes  ⬜ no | If yes, reason: | _____________________________________________________________  _____________________________________________________________ |
| --- | --- | --- |

**Mucositis**

⬜ None

⬜ Asymptomatic or mild symptoms; intervention not indicated

⬜ Moderate pain; not interfering with oral intake; modified diet indicated

⬜ Severe pain; interfering with oral intake

⬜ Life-threatening consequences; urgent intervention indicated

**Polyneuropathy of Taxol**

⬜ no

⬜ yes, ____________________________________________________________________________________

**Dysphagia according to the CTCAEE v. 4.03**

0, None

1, Symptomatic, able to eat regular diet

2, Symptomatic and altered eating/swallowing

3, Severely altered eating/swallowing; tube feeding or TPN or hospitalization indicated

4, Life-threatening consequences, urgent intervention indicated

**Dyspnea according to the CTCAE v. 4.03**

0, None

1, Shortness of breath with moderate exertion

2, Shortness of breath with minimal exertion; limiting instrumental ADL

3, Shortness of breath at rest; limiting self-care ADL

4, Life-threatening consequences, urgent intervention indicated
