# Supplementary material for: Feasibility study on pre or postoperative accelerated radiotherapy (POP-ART) in breast cancer patients
Source: Pilot Feasibility Stud. 2020 Oct 10;6:154. doi: 10.1186/s40814-020-00693-z (PMC7547514; doi:10.1186/s40814-020-00693-z)
Supplement: Supplementary file 1 — Additional file 1. Pre or postoperative accelerated radiotherapy (POP-ART) [file 40814_2020_693_MOESM1_ESM.zip › Additional file 1/POP-ART chemotherapy general EN.docx]

Pre or postoperative accelerated radiotherapy (POP-ART)

Neoadjuvant chemotherapy

Patient Initials ⬜ ⬜ ⬜ ⬜.

Date of Birth (dd/mm/yyyy) ⬜ ⬜ / ⬜ ⬜ / ⬜ ⬜ ⬜ ⬜

Date Completed (dd/mm/yyyyy) ⬜ ⬜ / ⬜ ⬜ / ⬜ ⬜ ⬜ ⬜

Name + Signature of Person completing the CRF __________________________________

**EC Dose Dense**

| Cycle 1 | date started: | ⬜ ⬜ / ⬜ ⬜ / ⬜ ⬜ ⬜ ⬜ |
| --- | --- | --- |
|  | if delayed: | number of days ⬜.  reason: _______________________________________________ |
| Cycle 2 | date started: | ⬜ ⬜ / ⬜ ⬜ / ⬜ ⬜ ⬜ ⬜ |
|  | if delayed: | number of days ⬜.  reason: _______________________________________________ |
| Cycle 3 | date started: | ⬜ ⬜ / ⬜ ⬜ / ⬜ ⬜ ⬜ ⬜ |
|  | if delayed: | number of days ⬜.  reason: _______________________________________________ |
| Cycle 4 | date started: | ⬜ ⬜ / ⬜ ⬜ / ⬜ ⬜ ⬜ ⬜ |
|  | if delayed: | number of days ⬜.  reason: _______________________________________________ |

**Taxol**

| Cycle 1 | date started: | ⬜ ⬜ / ⬜ ⬜ / ⬜ ⬜ ⬜ ⬜ |
| --- | --- | --- |
|  | if delayed: | number of days ⬜.  reason: _______________________________________________ |
| Cycle 2 | date started: | ⬜ ⬜ / ⬜ ⬜ / ⬜ ⬜ ⬜ ⬜ |
|  | if delayed: | number of days ⬜.  reason: _______________________________________________ |
| Cycle 3 | date started: | ⬜ ⬜ / ⬜ ⬜ / ⬜ ⬜ ⬜ ⬜ |
|  | if delayed: | number of days ⬜.  reason: _______________________________________________ |
| Cycle 4 | date started: | ⬜ ⬜ / ⬜ ⬜ / ⬜ ⬜ ⬜ ⬜ |
|  | if delayed: | number of days ⬜.  reason: _______________________________________________ |
| Cycle 5 | date started: | ⬜ ⬜ / ⬜ ⬜ / ⬜ ⬜ ⬜ ⬜ |
|  | if delayed: | number of days ⬜.  reason: _______________________________________________ |
| Cycle 6 | date started: | ⬜ ⬜ / ⬜ ⬜ / ⬜ ⬜ ⬜ ⬜ |
|  | if delayed: | number of days ⬜.  reason: _______________________________________________ |
| Cycle 7 | date started: | ⬜ ⬜ / ⬜ ⬜ / ⬜ ⬜ ⬜ ⬜ |
|  | if delayed: | number of days ⬜.  reason: _______________________________________________ |
| Cycle 8 | date started: | ⬜ ⬜ / ⬜ ⬜ / ⬜ ⬜ ⬜ ⬜ |
|  | if delayed: | number of days ⬜.  reason: _______________________________________________ |
| Cycle 9 | date started: | ⬜ ⬜ / ⬜ ⬜ / ⬜ ⬜ ⬜ ⬜ |
|  | if delayed: | number of days ⬜.  reason: _______________________________________________ |
| Cycle 10 | date started: | ⬜ ⬜ / ⬜ ⬜ / ⬜ ⬜ ⬜ ⬜ |
|  | if delayed: | number of days ⬜.  reason: _______________________________________________ |
| Cycle 11 | date started: | ⬜ ⬜ / ⬜ ⬜ / ⬜ ⬜ ⬜ ⬜ |
|  | if delayed: | number of days ⬜.  reason: _______________________________________________ |
| Cycle 12 | date started: | ⬜ ⬜ / ⬜ ⬜ / ⬜ ⬜ ⬜ ⬜ |
|  | if delayed: | number of days ⬜.  reason: _______________________________________________ |

**Herceptin SC, 18x**

|  | date started: | ⬜ ⬜ / ⬜ ⬜ / ⬜ ⬜ ⬜ ⬜ |
| --- | --- | --- |
|  | date of last dose: | ⬜ ⬜ / ⬜ ⬜ / ⬜ ⬜ ⬜ ⬜ |
|  | number of cycles: | ⬜ ⬜. |
|  | if < 18 cycles, reason: | ________________________________________ |
|  | interruption | ⬜ no  ⬜ yes, reason: ___________________________________________________ |
