# Supplementary material for: Feasibility study on pre or postoperative accelerated radiotherapy (POP-ART) in breast cancer patients
Source: Pilot Feasibility Stud. 2020 Oct 10;6:154. doi: 10.1186/s40814-020-00693-z (PMC7547514; doi:10.1186/s40814-020-00693-z)
Supplement: Supplementary file 1 — Additional file 1. Pre or postoperative accelerated radiotherapy (POP-ART) [file 40814_2020_693_MOESM1_ESM.zip › Additional file 1/POP-ART radiotherapy toxiciteit EN.docx]

Pre or postoperative accelerated radiotherapy (POP-ART)

CRF: Acute toxicity

Patient Initials ⬜ ⬜ ⬜ ⬜.

Date of Birth (dd/mm/yyyy) ⬜ ⬜ / ⬜ ⬜ / ⬜ ⬜ ⬜ ⬜

Date Completed (dd/mm/yyyyy) ⬜ ⬜ / ⬜ ⬜ / ⬜ ⬜ ⬜ ⬜

Name + Signature of Person completing the CRF __________________________________

| Registration moment^[[1]](#footnote-1)^ | 🞏 during RT | 🞏 end of RT | 🞏 2-4 weeks | 🞏 other |
| --- | --- | --- | --- | --- |

**Breast symptoms (side to be irradiated)**

**Pain**

⬜ none

⬜ only on contact

⬜ not only on contact, but occasionally

⬜ not only on contact and regularly

⬜ need for pain medication: _____________________________________________

**Sense of heaviness**

⬜ yes

⬜ no

**Itching**

⬜ none

⬜ occasionally

⬜ regularly

**Arm symptoms (side to be irradiated)**

**Pain**

⬜ none

⬜ occasionally

⬜ regularly

**Sense of heaviness**

⬜ yes

⬜ no

**Shoulder pain (side to be irradiated)**

**Pain**

⬜ none

⬜ occasionally

⬜ regularly

**Impaired mobility**

⬜ yes

⬜ no

**Pain other than breast, shoulder or arm pain**

⬜ none

⬜ occasionally

⬜ regularly

**Please indicate all painful localizations on the figure on the next page**


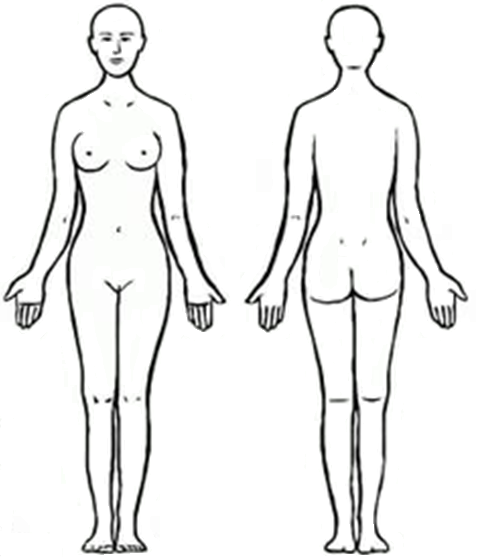


**Dysphagia according to the CTCAEE v. 4.03**

⬜ None

⬜ Symptomatic, able to eat regular diet

⬜ Symptomatic and altered eating/swallowing

⬜ Severely altered eating/swallowing; tube feeding or TPN or hospitalization indicated

⬜ Life-threatening consequences, urgent intervention indicated

**Dyspnea according to the CTCAE v. 4.03**

⬜ None

⬜ Shortness of breath with moderate exertion

⬜ Shortness of breath with minimal exertion; limiting instrumental ADL

⬜ Shortness of breath at rest; limiting self-care ADL

⬜ Life-threatening consequences, urgent intervention indicated

**Cough according to the CTCAE v. 4.03**

⬜ None

⬜ Mild symptoms, nonprescription intervention indicated

⬜ Moderate symptoms, medical intervention indicated; limiting instrumental ADL

⬜ Severe symptoms; limiting self-care ADL

**Fatigue according to the CTCAE v. 4.03**

⬜ None

⬜ Fatigue relieved by rest

⬜ Fatigue not relieved by rest, limiting instrumental ADL

⬜ Fatigue not relieved by rest, limiting self-care ADL

**Breast edema**

⬜ none

⬜ swelling or obscuration of anatomic architecture on close inspection

⬜ Readily apparent obscuration of anatomic architecture, obliteration of skin folds; readily apparent

deviation from normal anatomic contour, limiting instrumental ADL

⬜ Gross deviation from normal anatomic contour, limiting self-care ADL

**Dermatitis/desquamation according to the CTCAE v. 4.03**

0 = none

1 = Faint erythema or dry desquamation

2 = Moderate to brisk erythema; moderate edema; patchy moist desquamation, mostly confined to skin folds and creases^[[2]](#footnote-2)^

3 = Moist desquamation in areas other than skin folds and creases; bleeding induced by minor trauma or abrasion^2^

4 = Life-threatening consequences; skin necrosis or ulceration of full thickness dermis; spontaneous bleeding from involved site; skin graft indicated^2^


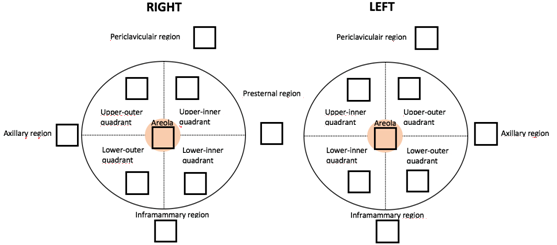


**Desquamation**

0 = None 1 = Dry desquamation 2 = Moist desquamation^2^

**
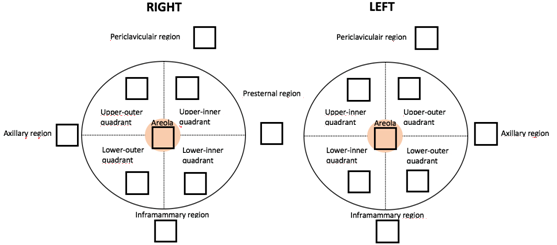
**

| **Arm circumference** | LEFT arm | RIGHT arm |
| --- | --- | --- |
| 15 cm above medial epicondyle | ⬜ ⬜, ⬜ cm | ⬜ ⬜, ⬜ cm |
| 15cm below medial epidcondyle | ⬜ ⬜, ⬜ cm | ⬜ ⬜, ⬜ cm |

**Troponine T value^[[3]](#footnote-3)^** ⬜ ⬜ , ⬜ pg/ml

1. At least 2 acute toxicity registrations are required: 1 evaluation at the last treatment session (+/- one day), and 1 evaluation at 8-16 days after treatment, as side-effects are most pronounced in this timespan. Toxicities that are scored at a later time can be registered. [↑](#footnote-ref-1)
2. Document with photographs: an overview of the upper body (without the head) with the hands resting on the hips and one with the arms above the head and detailed snapshots of the areas of greatest toxicity. [↑](#footnote-ref-2)
3. At baseline and the last treatment session (+/- 1 day) [↑](#footnote-ref-3)
