# Supplementary material for: Feasibility study on pre or postoperative accelerated radiotherapy (POP-ART) in breast cancer patients
Source: Pilot Feasibility Stud. 2020 Oct 10;6:154. doi: 10.1186/s40814-020-00693-z (PMC7547514; doi:10.1186/s40814-020-00693-z)
Supplement: Supplementary file 1 — Additional file 1. Pre or postoperative accelerated radiotherapy (POP-ART) [file 40814_2020_693_MOESM1_ESM.zip › Additional file 1/POP-ART surgery general EN.docx]

Pre or postoperative accelerated radiotherapy (POP-ART)

CRF: Surgery (general)

Patient initials ⬜ ⬜ ⬜ ⬜.

Birth date (dd/mm/yyyy) ⬜ ⬜ / ⬜ ⬜ / ⬜ ⬜ ⬜ ⬜

Date Completed (dd/mm/yyyy) ⬜ ⬜ / ⬜ ⬜ / ⬜ ⬜ ⬜ ⬜

Name + Signature of Person completing the CRF __________________________________

**Surgery + APD**

| surgery date (dd/mm/yyyy)  ⬜ ⬜/⬜ ⬜/⬜ ⬜ ⬜ ⬜ | type of surgery  ⬜ segment-/quadrantectomy  ⬜ wide local excision  ⬜ mastectomy  ⬜ not known |
| --- | --- |
| axillary surgery  ⬜ sentinel node biopsy  ⬜ axillary dissection  ⬜ sentinel node biopsy + axillary dissection | number of nodes involved ⬜.  number of nodes examined ⬜. |

**Effects of surgery**

**Perioperative**

| Blood loss ⬜ ⬜ ⬜ ⬜.⬜ml | operating time ⬜.⬜h⬜ ⬜ |
| --- | --- |
| Unexpected complications  ____________________________________________________________________________________________________________________________________________________________________ | |

**Immediately after surgery**

| number of additional surgeries | ⬜. | last surgery date (dd/mm/yyyy) | | | ⬜ ⬜/⬜ ⬜/⬜ ⬜ ⬜ ⬜ |
| --- | --- | --- | --- | --- | --- |
| hospitalization time first period (days) | ⬜. | pain | ⬜ none  ⬜ only on contact  ⬜ not only on contact, but occasionally  ⬜ not only on contact and regularly  ⬜ need for pain medication: ___________________________ | | |
| days of drainage | ⬜. | drainage | | ⬜.⬜ ⬜ ⬜ ml | |
| Hematoma/seroma | ⬜ Mild symptoms; intervention not indicated  ⬜ Minimally invasive evacuation or aspiration indicated  ⬜ Transfusion, radiologic, endoscopic, or elective operative intervention indicated  ⬜ Life-threatening consequences; urgent intervention indicated | | | | |

Total hospitalization time inclusive addition surgeries ⬜ ⬜.
