# Supplementary material for: Feasibility study on pre or postoperative accelerated radiotherapy (POP-ART) in breast cancer patients
Source: Pilot Feasibility Stud. 2020 Oct 10;6:154. doi: 10.1186/s40814-020-00693-z (PMC7547514; doi:10.1186/s40814-020-00693-z)
Supplement: Supplementary file 1 — Additional file 1. Pre or postoperative accelerated radiotherapy (POP-ART) [file 40814_2020_693_MOESM1_ESM.zip › Additional file 1/POP-ART surgery toxicity EN.docx]

Pre or postoperative accelerated radiotherapy (POP-ART)

CRF: Surgery toxicity

Patient initials ⬜ ⬜ ⬜ ⬜.

Birth date (dd/mm/yyyy) ⬜ ⬜ / ⬜ ⬜ / ⬜ ⬜ ⬜ ⬜

Date Completed (dd/mm/yyyy) ⬜ ⬜ / ⬜ ⬜ / ⬜ ⬜ ⬜ ⬜

Name + Signature of Person completing the CRF __________________________________

| registration moment | 🞏 | 🞏 | 🞏 |
| --- | --- | --- | --- |

**Effects of surgery**

**Woundhealing**

| Tissue | ⬜ healthy tissue  ⬜ necrotic tissue | Disease | ⬜ yes, ______________  ⬜ no |
| --- | --- | --- | --- |
| Moisture | ⬜ dry  ⬜ moist  ⬜ wet | healthy wound edge | ⬜ yes  ⬜ no |
| Infection/inflammation | ⬜ yes  ⬜ no | If yes: need of antibiotics | ⬜ yes  ⬜ no |

| abscess | ⬜ yes  ⬜ no | | | wick | ⬜ yes  ⬜ no | mastitis | ⬜ yes  ⬜ no |
| --- | --- | --- | --- | --- | --- | --- | --- |
|  |  | | |  |  |  |  |
| **breast edema** | | 0, none  1, swelling or obscuration of anatomic architecture on close inspection  2; Readily apparent obscuration of anatomic architecture, obliteration of skin folds; readily apparent deviation from normal anatomic contour, limiting instrumental ADL  3, Gross deviation from normal anatomic contour, limiting self-care ADL | | | | | |
|  | |  | | | | | |
| **seroma** | | ⬜ yes  ⬜ no | punction quantity  ⬜ ⬜.⬜l.⬜ ⬜ ⬜ ml | | | | |
| punction | | ⬜ yes  ⬜ no |  |  |  |  |  |
|  | |  |  | | | | |
| **pain** | | ⬜ none  ⬜ only on contact  ⬜ not only on contact, but occasionally  ⬜ not only on contact and regularly  ⬜ need for pain medication: __________________________________________________ | | | | | |

**Dermatitis/desquamation according to the CTCAE v. 4.03**

0 = none

1 = Faint erythema or dry desquamation

2 = Moderate to brisk erythema; moderate edema; patchy moist desquamation, mostly confined to skin folds and creases^[[1]](#footnote-1)^

3 = Moist desquamation in areas other than skin folds and creases; bleeding induced by minor trauma or abrasion^2^

4 = Life-threatening consequences; skin necrosis or ulceration of full thickness dermis; spontaneous bleeding from involved site; skin graft indicated^2^


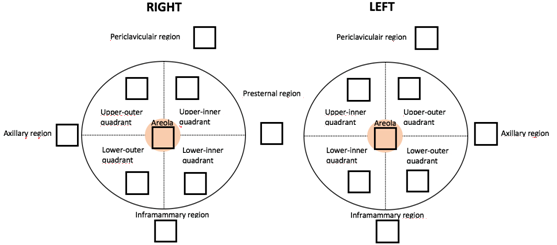


**Desquamation**

0 = None 1 = Dry desquamation 2 = Moist desquamation^2^

**
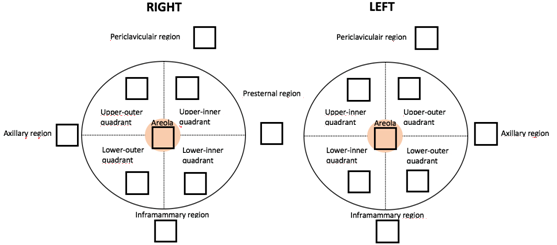
**

| **Arm circumference** | LEFT arm | RIGHT arm |
| --- | --- | --- |
| 15 cm above medial epicondyle | ⬜ ⬜, ⬜ cm | ⬜ ⬜, ⬜ cm |
| 15cm below medial epidcondyle | ⬜ ⬜, ⬜ cm | ⬜ ⬜, ⬜ cm |

**Brachial plexopathy^[[2]](#footnote-2)^**

| Some change in the sensitivity of the arm or hand (on the side to be irradiated) | ⬜ yes  ⬜ no |
| --- | --- |
| Deaf feeling or tingling in the hand or fingers (on the side to be irradiated) | ⬜ yes  ⬜ no |
| Problems with carrying or lifting objects with the arm (on the side to be irradiated) | ⬜ yes  ⬜ no |
| Problems with fingers such as writing or loosening a bottle cap (on the side to be irradiated) | ⬜ yes  ⬜ no |

**Brachial plexopathy, clinical examination:**

Flexion elbow

⬜ muscle can move joint against full resistance applied by examiner

⬜ muscle can move joint against mild/moderate resistance applied by examiner

⬜ muscle can move joint against gravity but without any resistance

⬜ muscle can move joint only if the force of gravity is eliminated

⬜ muscle contraction is seen or palpated but is insufficient to produce joint motion

⬜ no muscle contraction is seen or identified with palpation

Extension elbow

⬜ muscle can move joint against full resistance applied by examiner

⬜ muscle can move joint against mild/moderate resistance applied by examiner

⬜ muscle can move joint against gravity but without any resistance

⬜ muscle can move joint only if the force of gravity is eliminated

⬜ muscle contraction is seen or palpated but is insufficient to produce joint motion

⬜ no muscle contraction is seen or identified with palpation

Dorsiflexion wrist

⬜ muscle can move joint against full resistance applied by examiner

⬜ muscle can move joint against mild/moderate resistance applied by examiner

⬜ muscle can move joint against gravity but without any resistance

⬜ muscle can move joint only if the force of gravity is eliminated

⬜ muscle contraction is seen or palpated but is insufficient to produce joint motion

⬜ no muscle contraction is seen or identified with palpation

Plantar flexion wrist

⬜ muscle can move joint against full resistance applied by examiner

⬜ muscle can move joint against mild/moderate resistance applied by examiner

⬜ muscle can move joint against gravity but without any resistance

⬜ muscle can move joint only if the force of gravity is eliminated

⬜ muscle contraction is seen or palpated but is insufficient to produce joint motion

⬜ no muscle contraction is seen or identified with palpation

Shoulder abduction

⬜ muscle can move joint against full resistance applied by examiner

⬜ muscle can move joint against mild/moderate resistance applied by examiner

$$⬜ muscle can move joint against gravity but without any resistance

⬜ muscle can move joint only if the force of gravity is eliminated

⬜ muscle contraction is seen or palpated but is insufficient to produce joint motion

⬜ no muscle contraction is seen or identified with palpation

**Change in sensation (tested by gently rubbing)**

| 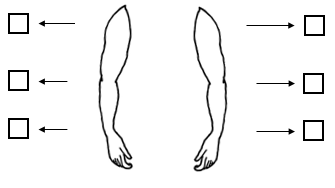 | 0 = no  1 = sensation different than the contralateral side  2 = no sensation at all |
| --- | --- |

1. Document with photographs: an overview of the upper body (without the head) with the hands resting on the hips and one with the arms above the head and detailed snapshots of the areas of greatest toxicity. [↑](#footnote-ref-1)
2. If brachial plexopathy is suspected, an electromyography should be performed [↑](#footnote-ref-2)
